# Phase I dose-escalation and pharmacokinetic study of a novel folate analogue AG2034

**DOI:** 10.1054/bjoc.2000.1601

**Published:** 2001-02

**Authors:** D Bissett, H L McLeod, B Sheedy, M Collier, Y Pithavala, L Paradiso, M Pitsiladis, J Cassidy

**Affiliations:** Department of Medicine and Therapeutics, University of Aberdeen, Aberdeen, UK; Agouron Pharmaceuticals Inc, La Jolla, California, USA; The Cancer Research Campaign, London, UK

**Keywords:** folate analogue, AG2034, GARFT inhibitor

## Abstract

The novel folate analogue AG2034, which was designed as an inhibitor of GARFT (glycinamide ribonucleotide formyltransferase), was evaluated in this phase I study under the auspices of The Cancer Research Campaign, UK. AG2034 blocks de novo purine synthesis through inhibition of GARFT. A total of 28 patients with histologically proven intractable cancers were enrolled. AG2034 was administered as a short intravenous infusion once every 3 weeks. 8 dose levels ranging from 1–11 mg/m^2^ were evaluated with patients receiving up to 6 cycles. Dose-limiting toxicities in the form of mucositis, diarrhoea and vomiting were observed at doses of 6 mg/m^2^ and above. Significant levels of thrombocytopenia, neutropenia and anaemia were also recorded. Other sporadic toxicities included fatigue and myalgia. The MTD with this schedule of AG2034 was 5 mg/m^2^. Most side effects occurred more frequently with cumulative dosing. In keeping with this, pharmacokinetic analysis revealed evidence of drug accumulation. The AG2034 AUC_0–24_ increased by a median of 184% (range 20–389%) from cycle 1 to 3 in all 10 patients examined. No objective antitumour responses were observed in the study. © 2001 Cancer Research Campaign http://www.bjcancer.com


*British Journal of Cancer* (2001) **84**(3), 308–312
© 2001 Cancer Research Campaign
doi: 10.1054/ bjoc.2000.1601, available online at http://www.idealibrary.com on IDEAL®

http://www.bjcancer.com

### Phase I dose-escalation and pharmacokinetic study of a novel folate analogue AG2034

**D Bissett[1], HL McLeod[1], B Sheedy[2], M Collier[2], Y Pithavala[2], L Paradiso[2], M Pitsiladis[3] and J Cassidy[1]**

[1]Department of Medicine and Therapeutics, University of Aberdeen, Aberdeen, UK; [2]Agouron Pharmaceuticals Inc, La Jolla, California, USA; [3]The Cancer Research Campaign, London, UK

**Summary** The novel folate analogue AG2034, which was designed as an inhibitor of GARFT (glycinamide ribonucleotide formyltransferase), was evaluated in this phase I study under the auspices of The Cancer Research Campaign, UK. AG2034 blocks de novo purine synthesis through inhibition of GARFT. A total of 28 patients with histologically proven intractable cancers were enrolled. AG2034 was administered as a short intravenous infusion once every 3 weeks. 8 dose levels ranging from 1–11 mg/m$^2$ were evaluated with patients receiving up to 6 cycles. Dose-limiting toxicities in the form of mucositis, diarrhoea and vomiting were observed at doses of 6 mg/m$^2$ and above. Significant levels of thrombocytopenia, neutropenia and anaemia were also recorded. Other sporadic toxicities included fatigue and myalgia. The MTD with this schedule of AG2034 was 5 mg/m$^2$. Most side effects occurred more frequently with cumulative dosing. In keeping with this, pharmacokinetic analysis revealed evidence of drug accumulation. The AG2034 AUC$_{0–24}$ increased by a median of 184% (range 20–389%) from cycle 1 to 3 in all 10 patients examined. No objective antitumour responses were observed in the study. © 2001 Cancer Research Campaign http://www.bjcancer.com

**Keywords**: folate analogue; AG2034; GARFT inhibitor

Glycinamide riboneucleotide formyltransferase (GARFT) is an essential enzyme in the pathway for de novo purine synthesis. Most normal tissues, with the exception of liver and activated T-lymphocytes, derive purines primarily through the salvage pathway. Tumour cells, in contrast, generally have elevated activity of the de novo pathway, and often have decreased activity of purine salvage enzymes, suggesting that they rely primarily upon de novo purine biosynthesis (Jackson and Harkrader, 1981). Selective inhibitors of purine biosynthesis may therefore have a different toxicity profile and possibly antitumour selectivity compared with other classes of antimetabolites.

The first selective GARFT inhibitor tested in clinical trials was (6R)-5,10-dideazatetrahydrofolate (lometrexol). This agent demonstrated objective antitumour activity in phase I studies but with unexpected toxicity, namely myelosuppression and mucositis (Ray et al, 1993). This was attributed to accumulation of polyglutamate metabolites in normal tissues and was ameliorated by the coadministration of either folic acid or folinic acid (Laohavinij et al, 1996; Sessa et al, 1996).

AG2034 is a novel and selective inhibitor of GARFT designed with knowledge of the X-ray crystal structures of the *E. coli* enzyme and of the GARFT domain of the human enzyme. Preclinical enzyme inhibition studies showed that AG2034 is a potent inhibitor of GARFT and a good substrate for folylpolyglutamate synthetase (FPGS), with similar potency to lometrexol. AG2034 can enter cells utilizing the reduced folate carrier and the membrane folate-binding protein. The agent has good antitumour activity in a broad range of tumour cell lines and human xenografts. Preclinical toxicological studies of AG2034 were conducted in mice and dogs. The major target organs for toxicity were the gastrointestinal tract and the bone marrow. Pre-treatment of animals with a diet deficient in folates enhanced these effects. When administered intravenously daily for 5 days the MTD of AG2034 was 0.2 mg kg$^{-1}$ day$^{-1}$ in mice fed a low-folate diet, compared with 40 mg kg$^{-1}$ day$^{-1}$ in mice fed a normal diet. Dogs were found to be relatively more sensitive than mice to the effects of AG2034. With the daily for 5 days schedule the no-effect-level (NOEL) was 0.2 mg kg$^{-1}$ day$^{-1}$ in dogs and 3 mg kg$^{-1}$ day$^{-1}$ in mice. With a single intravenous injection, the NOEL in dogs was 60 mg/m$^2$.

This phase I trial was initiated to evaluate AG2034 administered to patients with refractory solid malignancies. Although there was some evidence of schedule-dependent cytotoxicity in preclinical studies, in the interests of safety, a once every three weeks schedule was selected for this trial. The objectives of the study were (1) to evaluate the safety and dose tolerance of AG2034 when given by intravenous bolus injection to patients with advanced malignancy; (2) to study the pharmacokinetics and pharmacodynamics of AG2034; and (3) to document any antitumour effects of AG2034. Prior to commencing the study it was approved by the Grampian Health Board and University of Aberdeen Joint Ethical Committee.

*Received 19 May 2000*
*Revised 2 October 2000*
*Accepted 27 October 2000*

*Correspondence to:* D Bissett

### PATIENTS AND METHODS

### Eligibility criteria

Patients with histologically proven solid malignancy, for which no satisfactory treatment existed or against which established treatments had failed, were considered candidates for the study. Other eligibility criteria included: (1) WHO performance status 0, 1, or 2, (2) no prior chemotherapy within 4 weeks of study entry, (3) no radiotherapy, nitrosourea or mitomycin chemotherapy within 6 weeks of study entry, (4) satisfactory haematological and serum chemistry parameters, (5) age at least 18 years, (6) life expectancy of at least 3 months, and (7) written informed consent for the study. Patients were excluded from the trial if they had any of the following: (1) CNS disease which precluded informed consent, (2) severe co-existing medical condition, (3) evidence of bone marrow involvement by tumour or bone marrow compromise from previous anti-cancer therapy, (4) regular dietary folate supplements, (5) haematological malignancy, (6) concurrent medication with allopurinol or trimethoprim or other anticancer or experimental therapy, (7) prior therapy with a GARFT inhibitor, or (8) if they were pregnant, lactating, or unwilling to take reliable contraception if applicable.

### Treatment studies

This was an open label non-randomized phase I study with dose escalation between cohorts of patients. The study was conducted under the auspices of The Cancer Research Campaign, UK. Agouron Pharmaceuticals Inc., La Jolla, California, supplied AG2034, as a lyophilized powder, which was reconstituted with 4 ml water, resulting in a 5 mg $ml^{-1}$ solution. This was further diluted in 0.9% saline to a volume of 10 ml prior to administration as a 5 minute infusion. The starting dose of AG2034 was 1 $mg/m^2$, which was one sixtieth of the NOEL in dogs. Doses were repeated at 3-week intervals provided drug-related non-haematological toxicities had resolved and haematological parameters were satisfactory (Hb $\geq$ 10 g $dl^{-1}$, WCC $\geq 4.0 \times 10^9$ $l^{-1}$, and platelets $\geq 100 \times 10^9$ $l^{-1}$). Treatment was continued for a total of 6 cycles or until there was objective evidence of disease progression, or the development of toxicity precluding further therapy, or at the request of the patient. No antiemetic or other prophylactic medication was given with the first cycle of AG2034 but subsequently concurrent medication was administered as deemed appropriate by the clinician. No dose modifications were planned and dose escalation for individual patients was not permitted. There was no attempt to ameliorate toxicities with folate supplements or haematopoietic growth factors.

During the study patients were closely monitored, with weekly clinic visits for physical examination, toxicity evaluation, and blood sampling for full blood count and serum chemistry. Tumour assessment, usually by CT scan, was performed prior to study entry and after cycles 3 and 6 of AG2034.

A minimum of 3 patients were recruited to each dose level and if any of these patients experienced dose-limiting toxicity (DLT – see below for definition) a further 3 patients were treated at that dose level. Dose escalation was stopped when 2 or more members of a cohort experienced DLT. Dose escalation followed a modified Fibonacci scheme but was also guided by the toxicities observed in a concurrent phase I study of AG2034 conducted in the US using an identical schedule (Roberts et al, 2000).

### Tumour response and toxicity criteria

Tumour response was assessed according to the criteria of the CRC Phase I/II Trials Committee. Toxicities were graded according to the NCIC-CTG Expanded Common Toxicity Criteria. DLT was defined by the occurrence of any of the following: grade 4 neutropaenia or thrombocytopaenia, ≥ grade 3 anaemia, emesis uncontrolled by aggressive anti-emetic therapy, or other grade 3 non-haematological toxicities. The maximum tolerated dose was defined as the dose level associated with DLT in at least 2 of 6 patients.

### Pharmacokinetic study and analysis

Blood samples were collected from all patients for estimation of parent compound and metabolites. Samples were taken before drug administration in cycle one, and following drug administration at 5 and 30 minutes, at 1, 2, 4, 6, 8, 12, 24, 48, 72 and 96 hours, and weekly therafter. During the third cycle of AG2034, blood samples were taken at 5 and 30 minutes, and 1 and 24 hours after treatment. Subsequently weekly samples were taken until the patient was withdrawn from the trial. Plasma concentrations of AG2034 were measured using an ELISA assay (McLeod et al, 2000) and pharmacokinetic analyses were conducted using ADAPT II software (D'Argenio and Schumitzky, 1979).

### RESULTS

28 patients were enrolled into the study. Patient characteristics are listed in Table 1. The majority of patients were under the age of 65 years with good performance status. Metastatic colorectal cancer was the most frequent diagnosis, and while 24 patients had received prior chemotherapy only one patient had received more than 2 previous cytotoxic regimens. 20 patients had more than one site of disease.

All patients were assessable for toxicity. A total of 78 cycles of treatment were delivered over the dose range 1–11 $mg/m^2$, with a median of 3 cycles per patient (range 1–6). The treatment delivered is summarized in Table 2. Cohorts of patients were treated at escalating doses at 1, 1.5, 2.25, 3.4 and 5 $mg/m^2$. Recruitment of patients to the next two dose levels (7.5 and 11 $mg/m^2$) was stopped early following the occurrence of severe gastrointestinal toxicity at these dose levels in the parallel US phase I study of AG2034. In addition, preliminary analysis of pharmacokinetic data suggested accumulation of AG2034 through cycles 1–3. A cohort of 6 patients was then treated at the intermediate dose of 6 $mg/m^2$.

**Table 1** Patient characteristics

| | |
|---|---|
| Number of patients | 28 |
| Male : female | 18:10 |
| Age median | 59.5 |
| range | 34–76 |
| Performance status | |
| 0 | 1 |
| 1 | 24 |
| 2 | 3 |
| Primary tumour site | |
| Colorectal | 13 |
| Mesothelioma | 4 |
| Unknown primary | 2 |
| Ovary | 1 |
| Carcinoid | 1 |
| Cervix | 1 |
| Gallbladder | 1 |
| Hepatoma | 2 |
| Melanoma | 1 |
| Pancreas | 1 |
| Sarcoma | 1 |
| Previous treatment | |
| Chemotherapy | 24 |
| More than one chemotherapy regimen | 10 |
| Radiotherapy | 7 |

© 2001 Cancer Research Campaign 

**Table 2** Dose levels and number of cycles of AG2034 delivered

| Dose level (mg/m²) | Number of patients | Number of cycles | Actual dose of AG2034 Median (range) mg |
|---|---|---|---|
| 1 | 3 | 3, 4, 6 | 1.70 (1.50–2.20) |
| 1.5 | 3 | 3, 5, 3 | 2.50 (2.30–3.00) |
| 2.25 | 3 | 4, 3, 2 | 3.15 (2.85–3.94) |
| 3.4 | 4 | 1, 1, 2, 3 | 6.02 (5.50–6.30) |
| 5 | 3 | 3, 3, 3 | 9.40 (8.95–10.00) |
| 6 | 6 | 3, 1, 3, 3, 3, 3 | 11.19 (9.40–13.80) |
| 7.5 | 4 | 4, 1, 3, 3 | 15.75 (14.80–16.50) |
| 11 | 2 | 1, 1 | 21.73 (20.35–23.10) |

### Toxicities

The major toxicities observed are summarized in Tables 3 and 4. Gastrointestinal and haematological toxicities were reported at all dose levels. Stomatitis and diarrhoea that were dose limiting occurred in 2 out of 6 patients treated at 6 mg/m², defining this as the MTD. Stomatitis started 1–17 days after treatment with AG2034 (median 5 days) and resolved after 1–26 days (median 11.5 days). Diarrhoea started 1–21 days after treatment (median 6.5 days) and resolved after 1–17 days (median 8 days). Symptomatic treatment with loperamide was used throughout the study. There was evidence of cumulative gastrointestinal toxicity (Table 5). Grade 2 or worse mucositis was not seen until cycle 3 of AG2034 in 4 of the 5 affected patients, usually preceded by milder toxicity with the first two cycles. No attempt was made to modify these toxicities with folic acid supplements.

Haematological toxicities occurred sporadically throughout the study but no patients had dose-limiting myelosuppression (Table 4). The majority of patients experiencing thrombocytopenia or neutropenia received AG2034 at a dose of 5 mg/m² or more. There was no clear evidence of cumulative myelotoxicity during the study. Other minor toxicities occurred infrequently, including myalgia, neurosensory changes, and anorexia. Significant (grade 2) malaise and lethargy were reported by 4 of the 6 patients treated at 6 mg/m². Two patients had infections related to AG2034, one after cycle 2 at 7.5 mg/m², the other after cycle 1 at 11 mg/m². These were associated with only grade 1 neutropenia and both resolved with antibiotic therapy. One patient treated at the first dose level had grade 2 sensory peripheral neuropathy for several days after cycles 1 and 3. This patient had received two prior courses of chemotherapy, one of which included oxaliplatin. However no other patients in the study had similar symptoms although several patients had received prior platinum-based chemotherapy.

One patient had a dose delay (one week) and dose reduction (from 6 mg/m² to 5 mg/m²) because of grade 3 diarrhoea during cycle 2. This was done after consultation with the CRC and Agouron. Grade 3 diarrhoea recurred during cycle 3 and treatment was discontinued. Another patient had a treatment delay of 6 days because of grade 2 thrombocytopenia after cycle 2 (dose 7.5 mg/m²). Dose reductions were not applied in any other patients.

### Tumour response

No objective tumour responses were observed in the study. 18 patients had documented disease progression and two patients had stable disease during treatment with AG2034. Treatment was withdrawn in two cases because of unacceptable toxicity, two patients declined further treatment, one patient developed bowel

**Table 3** Gastrointestinal toxicities

| Dose (mg/m²) | Number of patients | Mucositis | | | Diarrhoea | | | Nausea | | | Vomiting | | |
|---|---|---|---|---|---|---|---|---|---|---|---|---|---|
| | | 2 | 3 | 4 | 2 | 3 | 4 | 2 | 3 | 4 | 2 | 3 | 4 |
| 1.0 | 3 | 0[a] | 0 | 0 | 0 | 0 | 0 | 0 | 0 | 0 | 1 | 0 | 0 |
| 1.5 | 3 | 0 | 0 | 0 | 0 | 0 | 0 | 0 | 0 | 0 | 0 | 0 | 0 |
| 2.25 | 3 | 0 | 0 | 0 | 1 | 0 | 0 | 0 | 0 | 0 | 0 | 0 | 0 |
| 3.4 | 4 | 0 | 0 | 0 | 0 | 0 | 0 | 1 | 0 | 0 | 0 | 0 | 0 |
| 5.0 | 3 | 0 | 0 | 0 | 0 | 0 | 0 | 1 | 0 | 0 | 1 | 0 | 0 |
| 6.0 | 6 | 1 | 1 | 1 | 1 | 1 | 0 | 1 | 0 | 0 | 1 | 0 | 0 |
| 7.5 | 4 | 1 | 1 | 0 | 0 | 0 | 0 | 1 | 0 | 0 | 1 | 1 | 0 |
| 11.0 | 2 | 0 | 0 | 0 | 1 | 0 | 0 | 2 | 0 | 0 | 0 | 1 | 0 |

[a]Data refer to the worst toxicity grade using the Expanded Common Toxicity Criteria (CTC), recorded for each patient at any cycle of AG2034.

**Table 4** Haematological toxicities

| Dose (mg/m²) | Number of patients | Neutropenia | | | Thrombocytopenia | | | Anaemia | | |
|---|---|---|---|---|---|---|---|---|---|---|
| | | 2 | 3 | 4 | 2 | 3 | 4 | 2 | 3 | 4 |
| 1.0 | 3 | 0[a] | 0 | 0 | 0 | 0 | 0 | 1 | 0 | 0 |
| 1.5 | 3 | 1 | 0 | 0 | 0 | 0 | 0 | 0 | 0 | 0 |
| 2.25 | 3 | 0 | 0 | 0 | 0 | 1 | 0 | 1 | 0 | 0 |
| 3.4 | 4 | 0 | 0 | 0 | 0 | 0 | 0 | 1 | 0 | 0 |
| 5.0 | 3 | 0 | 1 | 0 | 1 | 0 | 0 | 1 | 0 | 0 |
| 6.0 | 6 | 0 | 0 | 0 | 1 | 0 | 0 | 2 | 0 | 0 |
| 7.5 | 4 | 0 | 1 | 0 | 1 | 1 | 0 | 2 | 1 | 0 |
| 11.0 | 2 | 1 | 0 | 0 | 0 | 0 | 0 | 0 | 0 | 0 |

[a]Data refer to the worst CTC grade recorded for each patient at any cycle of AG2034.

*© 2001 Cancer Research Campaign*

**Table 5** Cumulative gastrointestinal toxicities

| | | | Stomatitis Cycle number | | | | | | Diarrhoea Cycle number | | | | | |
|---|---|---|---|---|---|---|---|---|---|---|---|---|---|---|
| **Dose level (mg/m²)** | **Number of cycles received** | **Number of cycles received** | **1** | **2** | **3** | **4** | **5** | **6** | **1** | **2** | **3** | **4** | **5** | **6** |
| 1.00 | 1 | 3 | | | | | | | | | | | | |
| | 2 | 4 | | | | | | | | | | | | |
| | 3 | 6 | | | | | | | | | | | | |
| 1.50 | 4 | 3 | | | | | | | | | | | | |
| | 5 | 5 | | | | G1 | G1 | | | G1 | | | | |
| | 6 | 3 | | | | | | | | | | | | |
| 2.25 | 7 | 4 | | | | G1 | | | | | | | | |
| | 8 | 3 | | | | | | | G2 | G1 | | | | |
| | 9 | 2 | | | | | | | | | | | | |
| 3.4 | 10 | 1 | | | | | | | | | | | | |
| | 11 | 1 | | | | | | | | | | | | |
| | 12 | 2 | | | | | | | | | | | | |
| | 13 | 3 | | | | | | | | | | | | |
| 5.00 | 14 | 3 | | | | | | | | | | | | |
| | 21 | 3 | | | | | | | | | | | | |
| | 22 | 3 | G1 | | G1 | | | | G1 | | | | | |
| 6.00 | 23 | 3 | | G3 | G4[a] | | | | | G1 | G2 | | | |
| | 24 | 1 | | | | | | | | | | | | |
| | 25 | 3 | G1 | G1 | G1 | | | | | | | | | |
| | 26 | 3 | | G1 | | | | | | G3 | G3 | | | |
| | 27 | 3 | G1 | G1 | G2 | | | | | | | | | |
| | 28 | 3 | | G1 | G3 | | | | G1 | | G1 | | | |
| 7.50 | 10 | 1 | G1 | G1 | G1 | G1 | | | | | G1 | | | |
| | 11 | 1 | G1 | | | | | | | | | | | |
| | 12 | 2 | G1 | G1 | G2[b] | | | | | | | | | |
| | 13 | 3 | G1 | G1 | G3 | | | | | | | | | |
| 11.00 | 18 | 1 | G1 | | | | | | | | | | | |
| | 19 | 1 | | | | | | | G2 | | | | | |

G = worst CTC toxicity Grade reported by the patient during this cycle. [a]Dose delayed by one week and reduced to 5 mg/m². [b]Dose delayed by one week because of thrombocytopenia.

**Table 6** Pharmacokinetic data

| Dose (mg/m²) | Number of patients | AUC cycle 1 AG2034 (μg ml min⁻¹)[a] |
|---|---|---|
| 1 | 3 | 8.2 (6.1–10.7) |
| 1.5 | 3 | 17.3 (16.7–19.7) |
| 2.25 | 3 | 38.3 (27.3–77.2) |
| 3.4 | 3 | 47.6 (42.2–78.6) |
| 5 | 3 | 143.8 (107.1–191.7) |
| 6 | 6 | 140.7 (115.4–255.1) |
| 7.5 | 2 | 95.3, 127.1 |
| 11 | 2 | 290.7, 323.2 |

[a]AUC data is presented as the median and range. Individual data are presented for the dose levels with only two data sets.

obstruction necessitating laparotomy, and another required palliative radiotherapy.

### Pharmacokinetics

AG2034 plasma concentrations were measured using an ELISA assay (McLeod et al, 2000). The assay has a linear range from 1–500 ng ml$^{-1}$ and an inter-assay coefficient of variation of 6.7–8.2%. Metabolites are not detected by the ELISA. AG2034 area under the concentration–time curve (AUC) was determined for each patient using the trapezoidal rule. Non-compartmental pharmacokinetic analysis was restricted to the first 24 hours after injection ($AUC_{0-24}$), to allow better comparison between different cycles of study drug. AG2034 pharmacokinetics were evaluable in 25 patients receiving 1–11 mg/m² as a bolus injection. AG2034 $AUC_{0-24}$ demonstrated a linear relationship with dose ($r^2 = 0.80$), with considerable variability in plasma drug exposure at each dose level (Table 6). There was evidence of drug accumulation, as the AG2034 $AUC_{0-24}$ increased from cycle 1 to 3 in 10/10 patients in whom samples were available for both cycles (median increase 184%, range 20% to 389%).

A more detailed pharmacokinetic and pharmacodynamic analysis of AG2034 has been produced by pooling data from this study with those from 29 patients treated in the US study (McLeod et al, 2000). Briefly the elimination of AG2034 was triphasic, with median values for $t_{1/2}\alpha$ 8.7 min, $t_{1/2}\beta$ 72.6 min, and $t_{1/2}\gamma$ 364.2 min. The systemic clearance of AG2034 ranged from 9.4–144.5 ml/min/m², and the volume of distribution was 1.2–7.6 litres/m².

### DISCUSSION

This report describes the first clinical experience with the GARFT inhibitor AG2034. As predicted from both preclinical data and previously reported clinical experience with lometrexol, the dose-limiting toxicities of this agent were stomatitis and diarrhoea.

*© 2001 Cancer Research Campaign*

These were accompanied by significant levels of myelosuppression, nausea and vomiting. For AG2034, as for lometrexol, cumulative toxicity appears to be a specific problem. However for AG2034 we have shown that this is associated with accumulation of the parent compound rather than toxic metabolites as in the case of lometrexol (Synold et al, 1998).

Although the pharmacokinetics of this agent showed large inter-patient variability, there was good correlation between dose and AUC for AG2034 during cycle one, implying that non-linearities in absorption or metabolism are not prominent during the first treatment cycle, at least at the doses used in this study. Previous studies with lometrexol have not provided pharmacokinetic evaluation beyond the first cycle of therapy (Wedge et al, 1995). This study demonstrates increase in the AG2034 $AUC_{0-24}$ over the three cycles evaluated. It suggests that altered AG2034 pharmacokinetics, with or without independent pharmacodynamic effects, are implicated in the cumulative toxicity observed with this agent.

An MTD of 5 mg/m$^2$ was established when AG2034 was given on a 3-weekly schedule of administration. Pharmacodynamic studies confirm the appropriateness of this dose. The estimated median AUC with 5 mg/m$^2$ is 131 580 ng ml$^{-1}$ min$^{-1}$ and in the range (+/- 10%) around this value no patients experienced toxicity. This contrasts with the 6 mg/m$^2$ AUC of 157 900 ng ml$^{-1}$ min$^{-1}$ in which range 2/4 patients had dose-limiting toxicity (McLeod, 2000). Further dose exploration would have included a more frequent schedule of administration, but drug accumulation precluded further study without folate supplementation to reduce toxicities. Future development of inhibitors to GARFT should focus on novel compounds which are more potent and selective, and which do not share problems of drug or metabolite accumulation.

### REFERENCES

D'Argenio DZ and Schumitzky A (1979) A program package for simulation and parameter estimation in pharmacokinetic systems. *Comput Prog Biomed* **9**: 115–134

Jackson RC and Harkrader RJ (1981) The contribution of *de novo* and salvage pathways of nucleotide biosynthesis in normal and malignant cells. In: *Nucleosides and Cancer Treatment*, Tattersall MHN, Fox RM (eds), pp 18–31. Sydney: Academic Press

Laohavinij S, Wedge SR, Lind MJ, Bailey N, Humphreys A, Proctor M, Chapman F, Simmons D, Oakley A, Robson L, Gumbrell L, Taylor GA, Thomas HD, Boddy AV, Newell DR and Calvert AH (1996) A phase I clinical study of the antipurine antifolate lometrexol (DDATHF) given with oral folic acid. *Invest New Drugs* **14**: 325–335

McLeod HL, Cassidy J, Powrie RH, Priest DG, Zorbas MA, Synold TW, Shibata S, Spicer D, Bissett D, Pithavala YK, Collier MA, Paradiso LJ and Roberts JD (2000) Pharmacokinetic and pharmacodynamic evaluation of the glycinamide ribonucleotide formyltransferase inhibitor AG2034. *Clin Cancer Res* **6**: 2677–2684

Ray MS, Muggia FM, Leichman CG, Grunberg SM, Nelson RL, Dyke RW and Moran RG (1993) Phase I study of (6R)-5,10-dideazatetrahydrofolate: a folate antimetabolite inhibitory to de novo purine synthesis. *J Natl Cancer Inst* **85**: 1154–1159.

Roberts JD, Shibata S, Spicer DV, McLeod HL, Tombes MB, Kyle B, Carroll M, Sheedy B, Collier MA, Pithavala YK, Paradiso LJ and Clendeninn NJ (2000). Phase I study of AG2034, a targeted GARFT inhibitor, administered once evry three weeks. *Cancer Chemother Pharmacol* **45**: 423–427.

Sessa C, de Jong J, D'Incalci M, Hatty S, Pagani O and Cavalli F (1996). Phase I study of the antipurine antifolate lometrexol (DDATHF) with folinic acid rescue. *Clin Cancer Res* **2**: 1123–1127

Synold TW, Newman EM, Carroll M, Muggia FM, Groshen S, Johnson K and Doroshow JH (1998) Cellular but not plasma pharmacokinetics of lometrexol correlate with the occurrence of cumulative haematological toxicity. *Clin Cancer Res* **4**: 2349–2355.

Wedge SR, Laohavinij S, Taylor GA, Boddy A, Calvert AH and Newell DR (1995) Clinical pharmacokinetics of the antipurine antifolate (6R)-5,10,dideaza-5,6,7,8-tetrahydrofolic acid (lometrexol) administered with an oral folic acid supplement. *Clin Cancer Res* **1**: 1479–1486.

*© 2001 Cancer Research Campaign*

## References

[PDF_00548] D'Argenio D. Z., Schumitzky A. (1979). A program package for simulation and parameter estimation in pharmacokinetic systems.. Comput Programs Biomed.

[PDF_00555] Laohavinij S., Wedge S. R., Lind M. J., Bailey N., Humphreys A., Proctor M., Chapman F., Simmons D., Oakley A., Robson L. (1996). A phase I clinical study of the antipurine antifolate lometrexol (DDATHF) given with oral folic acid.. Invest New Drugs.

[PDF_00560] McLeod H. L., Cassidy J., Powrie R. H., Priest D. G., Zorbas M. A., Synold T. W., Shibata S., Spicer D., Bissett D., Pithavala Y. K. (2000). Pharmacokinetic and pharmacodynamic evaluation of the glycinamide ribonucleotide formyltransferase inhibitor AG2034.. Clin Cancer Res.

[PDF_00565] Ray M. S., Muggia F. M., Leichman C. G., Grunberg S. M., Nelson R. L., Dyke R. W., Moran R. G. (1993). Phase I study of (6R)-5,10-dideazatetrahydrofolate: a folate antimetabolite inhibitory to de novo purine synthesis.. J Natl Cancer Inst.

[PDF_00569] Roberts J. D., Shibata S., Spicer D. V., McLeod H. L., Tombes M. B., Kyle B., Carroll M., Sheedy B., Collier M. A., Pithavala Y. K. (2000). Phase I study of AG2034, a targeted GARFT inhibitor, administered once every 3 weeks.. Cancer Chemother Pharmacol.

[PDF_00573] Sessa C., de Jong J., D'Incalci M., Hatty S., Pagani O., Cavalli F. (1996). Phase I study of the antipurine antifolate lometrexol (DDATHF) with folinic acid rescue.. Clin Cancer Res.

[PDF_00576] Synold T. W., Newman E. M., Carroll M., Muggia F. M., Groshen S., Johnson K., Doroshow J. H. (1998). Cellular but not plasma pharmacokinetics of lometrexol correlate with the occurrence of cumulative hematological toxicity.. Clin Cancer Res.

[PDF_00580] Wedge S. R., Laohavinij S., Taylor G. A., Boddy A., Calvert A. H., Newell D. R. (1995). Clinical pharmacokinetics of the antipurine antifolate (6R)-5,10- dideaza-5,6,7,8-tetrahydrofolic acid (Lometrexol) administered with an oral folic acid supplement.. Clin Cancer Res.

